# Changes in resting-state functional MRI connectivity during and after transcranial direct current stimulation in healthy adults

**DOI:** 10.3389/fnhum.2023.1229618

**Published:** 2023-07-20

**Authors:** Amy E. Bouchard, Emmanuelle Renauld, Shirley Fecteau

**Affiliations:** Department of Psychiatry and Neurosciences, Faculty of Medicine, CERVO Brain Research Centre, Centre intégré universitaire en santé et services sociaux de la Capitale-Nationale, Université Laval, Québec City, QC, Canada

**Keywords:** concurrent tDCS-fMRI, functional connectivity, DLPFC, healthy adults, parietal cortex

## Abstract

**Introduction:**

Transcranial direct current stimulation (tDCS) applied over the dorsolateral prefrontal cortex (DLPFC) at rest can influence behaviors. However, its mechanisms remain poorly understood. This study examined the effect of a single session of tDCS over the bilateral DLPFC on resting-state functional connectivity using fMRI (rs-fcMRI) during and after stimulation in healthy adults. We also investigated whether baseline rs-fcMRI predicted tDCS-induced changes in rs-fcMRI.

**Methods:**

This was a randomized, sham-controlled, double-blind, crossover study. We delivered tDCS for 30 min at 1 mA with the anode and cathode over the left and right DLPFC, respectively. We used seed-based analyses to measure tDCS-induced effects on whole-brain rs-fcMRI using a 3 (before, during, after stimulation) × 2 (active, sham stimulation) ANOVA.

**Results:**

There were four significant Time × Stimulation interactions on the connectivity scores with the left DLPFC seed (under the anode electrode) and no interactions for the right DLPFC seed (under the cathode electrode). tDCS changed rs-fcMRI between the left DLPFC seed and parieto-occipital, parietal, parieto-occipitotemporal, and frontal clusters during and after stimulation, as compared to sham. Furthermore, rs-fcMRI prior to stimulation predicted some of these tDCS-induced changes in rs-fcMRI during and after stimulation. For instance, rs-fcMRI of the fronto-parietooccipital network predicted changes observed after active stimulation, rs-fcMRI of the fronto-parietal network predicted changes during active stimulation, whereas rs-fcMRI of the fronto-parieto-occipitotemporal and the frontal networks predicted changes both during and after active stimulation.

**Discussion:**

Our findings reveal that tDCS modulated rs-fcMRI both during and after stimulation mainly in regions distal, but also in those proximal to the area under the anode electrode, which were predicted by rs-fcMRI prior to tDCS. It might be worth considering rs-fcMRI to optimize response to tDCS.

## 1. Introduction

In recent years, we have seen an exponential use of transcranial direct current stimulation (tDCS) in humans. This popularity mainly comes from the fact that tDCS is an easy, inexpensive, and non-invasive neuromodulatory technique with the potential to modulate brain activity that will, consequently, improve cognition, behaviors or alleviate symptoms. Unfortunately, the hasty use of tDCS has led to conflicting reports on the neural effects of tDCS in humans. True, the availability of tDCS devices that can be concurrently combined with neuroimaging is relatively recent and such investigations are not trivial to conduct. However, one remaining pitfall in the field is to take for granted the premises that anodal and cathodal tDCS, respectively, facilitates and inhibits cortical excitability, which is mainly based on the direct current stimulation literature, and that such local effects are causally related to the observed behavioral effects. Such premises derived from direct current stimulation to postulate mechanisms of tDCS are oversimplified and have created misleading data interpretations in the field of tDCS in humans (Jackson et al., 2016; Fecteau, 2022). Precise mechanisms of tDCS remain largely unknown (Bestmann and Walsh, 2017). This is unfortunate since a non-invasive approach that could reliably modify human brain activity would be impactful.

One fundamental need in the field of tDCS is to characterize and understand its effects on resting state brain activity. Indeed, little is still known on how tDCS influences brain activity, even when participants are simply at rest, without behavioral or cognitive confounding factors, such as performing cognitive tasks before, during or after stimulation. It seems important to investigate if tDCS alone reaches the cortex and sufficiently modulates brain activity. Characterization of the tDCS effects on resting state brain activity will contribute to developing hypotheses on how tDCS, by strengthening or weakening activity in brain regions and networks, may consequently improve cognitive performance. It will also contribute to identifying when it is relevant to deliver tDCS at rest, rather than combining it with a specific cognitive task. Further, characterization of tDCS effects during and after stimulation will contribute to identifying when it is best to combine it with cognitive tasks, whether they should be combined concurrently or subsequently.

Although tDCS delivered over the dorsolateral prefrontal cortex (DLPFC) is among the most used tDCS protocols in healthy individuals and patients with psychiatric conditions, there is still a paucity of studies on the effects of tDCS while delivered at rest on resting state functional connectivity (rs-fcMRI) using functional magnetic resonance imaging (fMRI), during and after stimulation. There seem to be only three studies that investigated the effects of tDCS at rest on rs-fcMRI after stimulation as compared to before stimulation. Keeser et al. (2011) conducted a double-blind, crossover tDCS study in 13 men who received tDCS with the anode and cathode over the left DLPFC and the right supraorbital area at 2 mA for 20 min. Rs-fcMRI was collected for 5 min while subjects had their eyes closed before and no later than 5 min after tDCS. Coactivation was increased in frontal regions, parts of the left frontal-parietal network and the right posterior cingulate cortex, as well as parts of the right frontal-parietal network after active than sham tDCS. Peña-Gómez et al. (2012) conducted a crossover, partially randomized (sham was always before active tDCS) study in 10 adults who received stimulation with the anode and cathode over the left DLPFC and right supraorbital area, respectively, and the opposite montage, at 2 mA for 20 min. Rs-fcMRI was collected for 10 min before and after tDCS. Temporal functional connectivity between prefrontal and parietal regions was stronger and spatial robustness of the default mode network was more reduced after active than after sham. Park et al. (2013) conducted a single-blind, parallel tDCS study, applying the anode over the left DLPFC and the cathode over the right supraorbital area at 1 mA for 20 min in healthy adults (25 in the active and 14 in the sham group). Rs-fcMRI was collected while subjects had their eyes closed immediately before and after tDCS. tDCS increased rs-fcMRI between the left DLPFC and frontal, temporal and subcortical regions in the right hemisphere and decreased it between the left DLPFC and frontal regions around the stimulation site in the left hemisphere.

There seem to be only two concurrent tDCS-fMRI studies reporting changes in rs-fcMRI in healthy humans while receiving tDCS over the DLPFC at rest. We previously conducted a sham-controlled, double-blind, crossover tDCS-fMRI study in 13 adults who received the anode and cathode electrodes over the left and right DLPFC, respectively, at 1 mA for 30 min (Mondino et al., 2020). Rs-fcMRI was collected for 5 min before tDCS, 30 min during tDCS and 10 min after tDCS while subjects had their eyes closed. Rs-fcMRI was increased between the left DLPFC seed under the anode electrode and bilateral parietal regions during stimulation, which long lasted for at least 10 min after stimulation. Leaver et al. (2022) conducted a single-blind, crossover tDCS-fMRI study in 37 adults who received 2 mA active and sham tDCS for 5 min, within the same session separated by 10–15 min. The anode and cathode were over the left DLPFC and right ventrolateral PFC, respectively. During active as compared to sham stimulation, rs-fcMRI increased within the orbitofrontal network, whereas it decreased between a frontoparietal network and a node near the subgenual anterior cingulate cortex, as well as a node near the right superior parietal lobule.

In sum, the number of studies that delivered tDCS over the DLPFC at rest and measured rs-fcMRI, without any confounding factors such as administering a task before, during and or after stimulation, remains limited. Although results between studies still show some variability, the main effects indicate modulation of large-scale circuits involving proximal and distal regions to the DLPFC (especially frontal and parietal areas).

The main goal of this work was to examine the tDCS effects on rs-fcMRI delivered over the DLPFC while healthy adults were at rest, and if so, whether baseline rs-fcMRI predicts such effects. Specifically, we investigated (1) the type of changes induced by tDCS (i.e., increases rs-fcMRI, further positively correlates brain regions, decreases rs-fcMRI, further anticorrelates brain regions); (2) the location of these changes (i.e., between the whole brain and each region under the anode and cathode electrodes, the left and right DLPFC); (3) the time course of these changes (during and/or after stimulation); and (4) if baseline rs-fcMRI predicts these changes, if any. We based our hypotheses on the study by Mondino et al. (2020) since it seems to be the only study that investigated tDCS effects on rs-fcMRI while targeting the bilateral DLPFC in healthy adults at rest both during and after stimulation. We expected that tDCS will increase fronto-parietal rs-fcMRI, both during and after stimulation.

## 2. Materials and methods

### 2.1. Design

This was a randomized, crossover, sham-controlled, double-blind study. Participants underwent two concurrent tDCS-fMRI sessions, one with active and one with sham tDCS, separated by 7 days (on the same weekday and time of day to minimize variability). They were randomized using a Latin square, counterbalancing the order of active and sham tDCS. Blinding integrity was assessed in participants and the outcome assessor using a standardized form to determine whether they believed the session was active or sham tDCS. Participants were also assessed on potential tDCS-related side effects at each tDCS session using a standardized questionnaire.

### 2.2. Participants

Sixteen healthy participants enrolled in this project. They were free of general medical, neurological, and psychiatric conditions and eligible for tDCS (Keel et al., 2001) and MRI. They provided their written informed consent prior to their participation in this study. The institutional review board of the local institute approved this project. Fourteen participants completed the study (two participants withdrew). We excluded one participant due to MRI artifacts. Thus, 13 participants (nine women; mean age = 26.1, standard deviation = 4.6 years; one left-handed, one ambidextrous, eleven right-handed evaluated using the Edinburgh Handedness Inventory, Oldfield, 1971) were entered into the analyses. A sample of 13 participants was needed to detect a large effect size (dz = 0.85) with 80% power and α of 0.05, for a two-tailed paired samples *t*-test (Faul et al., 2007, 2009).

### 2.3. tDCS

We administered tDCS using an MR-compatible battery-driven stimulator (neuroConn GmbH, Ilmenau, Germany) with two 7 × 5 cm^2^ rubber electrodes. We used an electrode paste (≈3 mm layer) to offer stability (e.g., less chance to drip and bridge between the electrodes than saline water) and prevent drying out over the scanning session. Active tDCS was delivered at a current intensity of 1 mA (maximum current intensity applicable for the stimulator used in this study) for 30 min. Sham stimulation was delivered for 30 min with ramp up and ramp down periods of 30 s, the remaining time with no active current (Gandiga et al., 2006). The anode and cathode electrodes were placed over the left (F3) and right (F4) DLPFC, respectively, using the international electroencephalography 10–20 system. We chose to apply the anode and cathode electrodes over the left and right DLPFC, respectively, since this montage previously led to results of interest in our research program on substance use disorders. We observed that this montage modulated decision-making behaviors relevant for substance use disorders (Fecteau et al., 2010), such as reducing risk taking behaviors (Fecteau et al., 2007) and elevation of salivary cortisol during decision making under stress condition (Brunelin and Fecteau, 2021). This montage also reduced cue-provoked craving for alcohol (Boggio et al., 2008), smoking (Boggio et al., 2009), and food (Fregni et al., 2008; see Bouchard et al., 2021 for a review). We now pursue investigation of this montage by combining it concurrently with neuroimaging. So far, we found that this montage elevated prefrontal N-acetylaspartate and striatal glutamate + glutamine (Hone-Blanchet et al., 2016) and rs-fcMRI between the left DLPFC and bilateral parietal regions (Mondino et al., 2020). Our ultimate goal is to identify the mechanisms of this montage to eventually offer a neuromodulatory method that will reliably engage specific brain targets.

### 2.4. MRI

#### 2.4.1. Data acquisition

We acquired data as follows: 5 min of fMRI before tDCS, 25 min of fMRI during tDCS (onset of fMRI acquisition was 5 min after the start of stimulation), 5 min of fMRI after tDCS, and the anatomical scan. For the fMRI scans, we instructed participants to rest and keep their eyes open. Whole-brain MR scans were acquired with a Philips 3T Achieva scanner and a standard 8-channel head coil (Philips Healthcare, Best, Netherlands). T1-weighted structural magnetic images were obtained with a magnetization prepared rapid acquisition gradient-echo sequence with the following parameters: TR = 8.2 ms, TE = 3.7 ms, FoV = 250 mm, flip angle = 8°, 256 × 256 matrix, 180 slices/volume, slice thickness = 1 mm, no gap. For the rs-fcMRI scans, EPI BOLD images were acquired as follows: TR = 3,000 ms, TE = 30 ms, FoV = 224 mm × 224 mm × 140 mm, flip angle = 70°, 64 × 64 matrix, dynamic scans 100, voxel size = 3.5 mm × 3.5 mm × 3.5 mm, slice thickness = 3.5 mm, no gap.

#### 2.4.2. fMRI preprocessing

We preprocessed structural and functional volumes with CONN (Whitfield-Gabrieli and Nieto-Castanon, 2012) (version 19.c) and SPM 12 on MATLAB R2019a (Mathworks, Inc., USA). We used CONN’s default preprocessing pipeline (Nieto-Castanon, 2020). We smoothed volumes with 7 mm full width at half Gaussian kernel. We used ART^1^ to identify outlier scans with intermediate settings (97th percentile in normative sample). We defined outliers using a global signal z-value threshold of 5 and a subject-motion mm threshold of 0.9 mm. We excluded one participant because he had more than 20% outlier scans. We denoised data using Compcor (Behzadi et al., 2007) to regress out physiological noise sources (white matter and cerebrospinal fluid signals with 10 confound dimensions in addition to their first-order derivatives). Moreover, we regressed out movement-related covariates (scrubbing and realignment, with its first-order derivatives). We also regressed out the effect of each session (before, during and after tDCS), with their first-order derivatives. We performed band pass filtering of 0.008–0.09 Hz (Hallquist et al., 2013) and linear detrending. Lastly, we verified preprocessing and denoising procedures with CONN’s quality analysis reports. We labeled cortical and subcortical regions using the Harvard-Oxford Atlas (Desikan et al., 2006) and cerebellar areas with the automated anatomical labeling atlas (Tzourio-Mazoyer et al., 2002), as implemented in CONN.

#### 2.4.3. Seed-based rs-fcMRI analyses

We conducted seed-based rs-fcMRI analyses with the left and right DLPFC as seeds (*x* = ± 36, *y* = 29, *z* = 38; with 5 mm radii) using CONN. These analyses measure the level of rs-fcMRI between the seed and each voxel in the brain (Nieto-Castanon, 2020). We performed a 3 × 2 (Time × Stimulation) repeated-measures ANOVA to investigate potential tDCS-induced changes on rs-fcMRI. We used a voxel threshold of *p*-uncorrected < 0.001 and cluster threshold cluster size of *p*-FDR-corrected < 0.05 (Friston et al., 1994). We calculated average connectivity values within the cluster(s) with REX,^2^ as implemented in CONN. We used SPSS 29 (IBM Corp., USA) to conduct *post hoc* analyses. We performed linear regression analyses to investigate whether baseline rs-fcMRI predicted tDCS-induced changes in rs-fcMRI of the significant clusters. *Post hoc* and linear regression analyses were bootstrapped with 1,000 bootstrap samples and 95% confidence intervals to confirm robustness.

## 3. Results

### 3.1. Effects of tDCS on rs-fcMRI during and after stimulation

**FIGURE 1:**
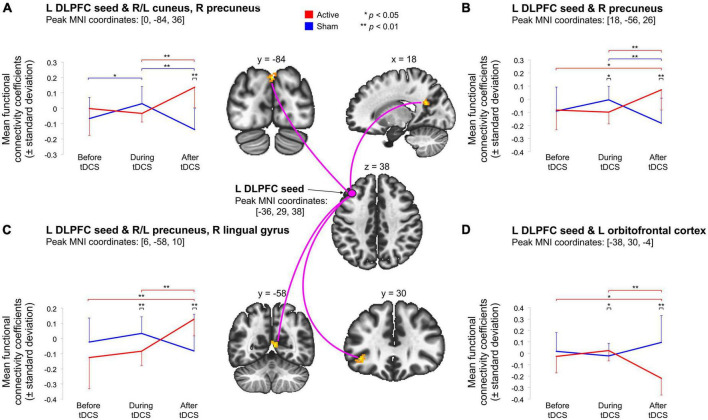
Illustration of the significant Time (pre-tDCS, during tDCS-post-tDCS) × Stimulation (active, sham) interactions indicate that active tDCS modulated rs-fcMRI between the left DLPFC seed (under the anodal electrode) and four clusters, during or after stimulation. First, **(A)** shows that active tDCS modulated rs-fcMRI between the left DLPFC seed and a parieto-occipital cluster. Second, **(B)** demonstrates that active tDCS modulated rs-fcMRI between the left DLPFC seed and a parietal cluster. Third, **(C)** indicates that active tDCS modulated rs-fcMRI between the left DLPFC seed and a parieto-occipitotemporal cluster. Last, **(D)** shows that active tDCS modulated rs-fcMRI between the left DLPFC seed and a frontal cluster. Voxel-threshold of *p* < 0.001 (*p*-uncorrected); cluster threshold of *p* < 0.05 (*p*-FDR-corrected). The red and blue error bars represent within-group differences for the active and sham condition, respectively. Asterisks denote significant *post hoc* comparisons (*p < 0.05; **p < 0.01).

We first compared baseline rs-fcMRI between active and sham conditions and found no differences for both seeds (left DLPFC: *p*-FDR = 0.540; right DLPFC: *p*-FDR ≥ 0.282). We then assessed the effects of tDCS on rs-fcMRI. Seed-based analyses for the left DLPFC seed (under the anode electrode) revealed four significant Time × Stimulation interactions (Table 1 and Figure 1). First, rs-fcMRI changed between the left DLPFC seed and a cluster mainly containing the bilateral cuneus (Figure 1A). Rs-fcMRI was greater (positively correlated) post-tDCS as compared to during tDCS and post-sham. Also, rs-fcMRI increased (changed from an anticorrelation to a positive correlation) from pre-sham to during sham, and then decreased (anticorrelated) from during sham to post-sham. Second, rs-fcMRI changed between the left DLPFC seed and a cluster in the right precuneus (Figure 1B). Rs-fcMRI was weaker during active than during sham, but greater post-tDCS as compared to pre-tDCS, during tDCS and post-sham (changed from an anticorrelation to a positive correlation). Also, rs-fcMRI was weaker post-sham as compared to during sham. Third, rs-fcMRI changed between the left DLPFC seed and a cluster mainly encompassing the bilateral precuneus, extending to the right lingual gyrus (Figure 1C). Rs-fcMRI was weaker during active than sham, and greater (changed from an anticorrelation to a positive correlation) post-tDCS as compared to pre-tDCS, during tDCS and post-sham. Fourth, rs-fcMRI changed between the left DLPFC seed and a cluster in the left orbitofrontal cortex (OFC) (Figure 1D). Rs-fcMRI was greater during active than sham, and weaker post-tDCS as compared to pre-tDCS, during tDCS and post-sham. There were no significant Time × Stimulation interactions on rs-fcMRI for the right DLPFC seed (under the cathode electrode; *p*-FDR = 0.584).

**TABLE 1:** tDCS-induced rs-fcMRI changes in healthy individuals revealed by time (before, during, after tDCS) × tDCS (active, sham) repeated measures ANOVA (significant *post hoc* results are bolded).

Left dorsolateral prefrontal cortex seed [*x* = −36, *y* = 29, *z* = 38]			
Right/Left cuneus, right precuneus			
Cluster size: 99 voxels	Peak MNI coordinates: [*x* = 0, *y* = −84, *z* = 36]		Size *p*-FDR = 0.0009
*Post hoc* comparison *t*-tests	*t*	*p*	Glass’ Δ
Before active vs. sham	−1.647	0.132	
During active vs. sham	2.071	0.071	
After active vs. sham	−5.968	**0.001**	
Before vs. during sham	−2.644	**0.033**	0.701
Before vs. during active	0.624	0.527	−0.182
Before vs. after sham	1.736	0.126	−0.520
Before vs. after active	−2.183	0.065	0.793
During vs. after sham	4.382	**0.004**	−1.497
During vs. after active	−4.156	**0.008**	3.003
**Right precuneus**			
**Cluster size: 55 voxels**	**Peak MNI coordinates: [*x* = 18, *y* = −56, *z* = 26]**		**Size *p*-FDR = 0.0186**
Before active vs. sham	−0.166	0.859	
During active vs. sham	3.163	**0.013**	
After active vs. sham	−5.563	**0.003**	
Before vs. during sham	−1.737	0.109	0.473
Before vs. during active	0.364	0.738	−0.107
Before vs. after sham	1.612	0.122	−0.492
Before vs. after active	−3.443	**0.021**	1.016
During vs. after sham	5.066	**0.002**	−1.751
During vs. after active	−4.434	**0.006**	1.899
**Right/Left precuneus, right lingual gyrus**			
**Cluster size: 42 voxels**	**Peak MNI coordinates: [*x* = 6, *y* = −58, *z* = 10]**		**Size *p*-FDR = 0.0438**
Before active vs. sham	1.576	0.137	
During active vs. sham	3.704	**0.004**	
After active vs. sham	−3.880	**0.005**	
Before vs. during sham	−1.426	0.185	0.365
Before vs. during active	−0.621	0.552	0.201
Before vs. after sham	0.864	0.387	−0.363
Before vs. after active	−4.137	**0.008**	1.229
During vs. after sham	1.805	0.110	−1.065
During vs. after active	−5.544	**0.001**	2.236
**Left orbitofrontal cortex**			
**Cluster size: 38 voxels**	**Peak MNI coordinates: [*x* = −38, *y* = 30, *z* = −4]**		**Size *p*-FDR = 0.0496**
Before active vs. sham	0.784	0.448	
During active vs. sham	−2.243	**0.048**	
After active vs. sham	5.792	**0.002**	
Before vs. during sham	0.795	0.418	−0.241
Before vs. during active	−1.708	0.097	0.360
Before vs. after sham	−1.029	0.337	0.477
Before vs. after active	2.855	**0.020**	−1.346
During vs. after sham	−1.813	0.097	1.072
During vs. after active	4.681	**0.001**	−2.692
**Right dorsolateral prefrontal cortex seed [*x* = 36, *y* = 29, *z* = 38]**			**Size *p*-FDR = 0.5844**

FDR, false discovery rate; MNI, Montreal Neurological Institute. *P*-values for *post hoc* comparison *t*-tests are bootstrapped.

## 4. Impact of baseline rs-fcMRI on tDCS effects during and after stimulation

**FIGURE 2:**
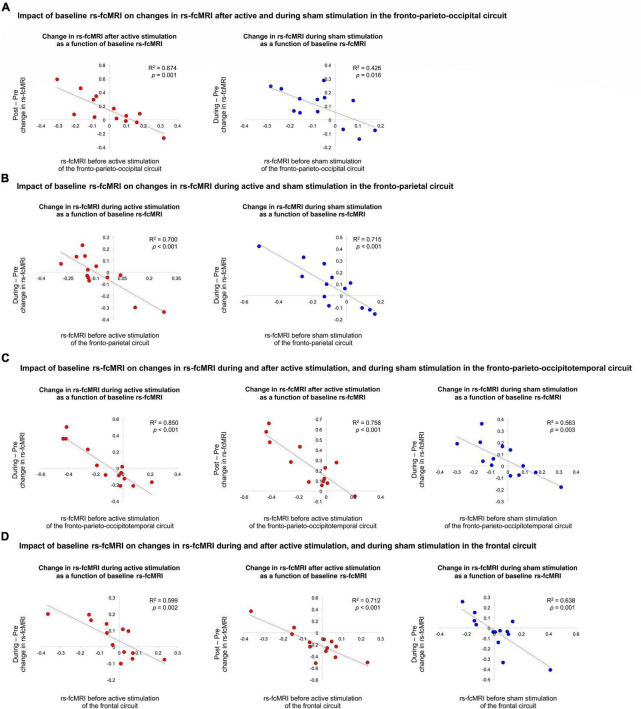
Impact of baseline rs-fcMRI on changes in rs-fcMRI during and after active and sham stimulation. First, **(A)** illustrates the impact of baseline rs-fcMRI on changes in rs-fcMRI after active and during sham stimulation in the fronto-parieto-occipital circuit. Second, **(B)** demonstrates the impact of baseline rs-fcMRI on changes in rs-fcMRI during active and sham stimulation in the fronto-parietal circuit. Third, **(C)** shows the impact of baseline rs-fcMRI on changes in rs-fcMRI during and after active stimulation, as well as during sham stimulation, in the fronto-parieto-occipitotemporal circuit. Last, **(D)** illustrates the impact of baseline rs-fcMRI on changes in rs-fcMRI during and after active stimulation, as well as during sham stimulation, in the frontal circuit. The red and blue trendlines and data points represent the active and sham conditions, respectively.

We then examined if rs-fcMRI prior to tDCS predicted rs-fcMRI changes in the four significant Time × Stimulation interactions involving the left DLPFC seed, under the anode electrode. We conducted regression analyses with baseline rs-fcMRI as the predictor and tDCS-induced changes in rs-fcMRI as the criterion variable [Bonferroni threshold *p* ≤ 0.05/15 = 0.00333: 3 comparisons for the first interaction (after active, during sham, after sham), 4 comparisons for each of the three other interactions (during and after active, during and after sham), totaling 15, Table 2 and Figure 2]. For the first interaction, baseline rs-fcMRI between the left DLPFC seed and the cluster mainly containing the bilateral cuneus predicted changes after tDCS (*p* = 0.001), accounting for 67.4% (*R*^2^ = 0.674) of the variance, but did not significantly predict changes during or after sham (during: *p* = 0.016; after: *p* = 0.051). For the second interaction, baseline rs-fcMRI between the left DLPFC seed and the right precuneus predicted changes during active stimulation (*p* < 0.001), which accounted for 70.0% (*R*^2^ = 0.700) of the variance. Baseline rs-fcMRI did not predict changes after active (*p* = 0.073) or sham stimulation (*p* = 0.068). For the third interaction, baseline rs-fcMRI between the left DLPFC seed and the cluster mainly containing the bilateral precuneus, extending to the right lingual gyrus, predicted changes during (*p* < 0.001) and after (*p* < 0.001) tDCS, accounting for 85.0% (*R*^2^ = 0.850) and 75.8% (*R*^2^ = 0.758) of the variance, respectively. Finally, for the fourth interaction, baseline rs-fcMRI between the left DLPFC seed and the cluster in the left OFC predicted changes during (*p* = 0.002) and after (*p* < 0.001) tDCS, accounting for 59.9% (*R*^2^ = 0.599) and 71.2% (*R*^2^ = 0.712) of the variance, respectively.

**TABLE 2:** Predictions from baseline rs-fcMRI (immediately before tDCS) on subsequent tDCS-induced rs-fcMRI changes in healthy individuals during and after stimulation between the left DLPFC seed and the fronto-parieto-occipital, fronto-parietal, fronto-parieto-occipitotemporal, and frontal circuits (significant *post hoc* results are bolded).

					Bootstrap	
	β	*R* ^2^	*t*	*p*	Bca 95% CI	*p*
**Right/Left cuneus, right precuneus**						
After active	−0.821	0.674	−4.767	**0.001**	[−1.442, −0.531]	**0.020**
During sham	−0.653	0.426	−2.860	**0.016**	[−0.959, −0.0506]	**0.019**
After sham	−0.551	0.303	−2.187	0.051	[−0.990, −0.272]	**0.013**
**Right precuneus**						
During active	−0.837	0.700	−5.070	**< 0.001**	[−1.217, −0.515]	**0.002**
After active	−0.512	0.262	−1.978	0.073	[−1.539, 0.244]	0.306
During sham	−0.846	0.715	−5.253	**< 0.001**	[−1.104, −0.565]	**0.001**
After sham	−0.520	0.271	−2.020	0.068	[−0.860, −0.208]	**0.008**
**Right/Left precuneus, right lingual gyrus**						
During active	−0.922	0.850	−7.888	**< 0.001**	[−1.381, −0.827]	**0.001**
After active	−0.871	0.758	−5.878	**< 0.001**	[−1.270, −0.559]	**0.002**
During sham	−0.750	0.563	−3.765	**0.003**	[−1.282, −0.293]	**0.001**
After sham	−0.332	0.110	−1.166	0.268	[−1.193, 0.670]	0.132
**Left orbitofrontal cortex**						
During active	−0.774	0.599	−4.050	**0.002**	[−1.085, −0.427]	**0.006**
After active	−0.844	0.712	−5.217	**< 0.001**	[−1.725, −0.851]	**0.001**
During sham	−0.799	0.638	−4.401	**0.001**	[−1.385, −0.248]	**0.002**
After sham	−0.526	0.276	−2.050	0.065	[−1.789, 0.789]	0.256

Bca 95% CI, bias corrected accelerated 95% confidence interval.

### 4.1. Side effects and integrity of blinding

There were no differences between active and sham tDCS for the number and intensity level of reported side effects (*p* > 0.1), nor mood (*p* > 0.4). There was no significant difference in blinding ratings between active and sham conditions (*p* > 0.7). The majority of participants were blind to the stimulation conditions they received (4 participants correctly guessed their stimulation condition with a confidence level higher than 90%). The rs-fcMRI assessor had minimal interaction with the participants and stayed blinded to the stimulation conditions with a 100% confidence level.

## 5. Discussion

In this sham-controlled, double-blind, crossover study, 30 min of tDCS delivered while healthy adults were at rest, with the anode and cathode electrode over the left and right DLPFC, respectively, modulated rs-fcMRI. Briefly, regarding our four specific aims, tDCS induced rs-fcMRI changes: (1) leading to increased (further positively correlated) and decreased (further negatively correlated or anticorrelated) rs-fcMRI, (2) in regions proximal and distal to the anode electrode, (3) in the same direction during and after stimulation (i.e., a change observed during stimulation was further increased after stimulation rather than reverted), with stronger changes after than during stimulation, and (4) some changes were predicted by baseline rs-fcMRI.

There were four significant stimulation (active, sham) by time (before, during, after tDCS) interactions. Interestingly, all rs-fcMRI changes were found with the left DLPFC seed, under the anode electrode, and none were found with the right DLPFC seed, under the cathode electrode.

Three out of the four stimulation by time interactions involved clusters contralateral and distal to the anode electrode. These interactions indicated rs-fcMRI changes between the left DLPFC seed and parietal, occipitoparietal and parieto-occipitotemporal networks. Further, baseline rs-fcMRI predicted some of these changes either during or after stimulation. These predictions indicate that it may be worth selecting participants based on their rs-fcMRI to optimize tDCS response. For instance, participants with stronger rs-fcMRI of the fronto-parietal network would respond better to tDCS during stimulation, whereas those with stronger rs-fcMRI in the fronto-parietooccipital network would respond better to tDCS after stimulation. This also might help improve study designs, such as identifying the best time point to test outcomes, whether during or after stimulation.

Most previous studies also reported that tDCS over the DLPFC increases rs-fcMRI in fronto-parietal networks, involving various parietal nodes (Keeser et al., 2011; Peña-Gómez et al., 2012; Mondino et al., 2020; Leaver et al., 2022). One also found strengthened rs-fcMRI in fronto-temporal networks (Park et al., 2013). In regard to modulating rs-fcMRI between frontal and parietooccipital nodes, it seems that this study might be the first one to report such a result. Generally, the prefrontal cortex in these large resting state networks presumably exerts top-down control of these parietal, temporal, and occipital nodes. It is still unclear in the rs-fcMRI literature how to interpret such tDCS-induced modulation leading these large networks to decorrelate. Intrinsic anticorrelations are observed between regions involved in externally oriented (e.g., attention) and internally oriented (e.g., self-referential processing) functions, possibly reflecting the separation of regions/networks with opposing or competing roles (Fox et al., 2005; Fox and Raichle, 2007; Buckner et al., 2008; Buckner and DiNicola, 2019), and possibly the capacity to switch between them (Whitfield-Gabrieli and Ford, 2012).

Interestingly, the fourth stimulation by time interaction differed from the three other interactions, that is rs-fcMRI change involved a cluster proximal and ipsilateral to the anode electrode. This change was observed between the left DLPFC and OFC. It indicated that active tDCS further anticorrelated rs-fcMRI of these regions after stimulation, as compared to before and during stimulation. Also, rs-fcMRI was greater during active than sham stimulation. Further, these changes during and after active stimulation were predicted by baseline rs-fcMRI of this frontal network. Park et al. (2013) found decreased rs-fcMRI in the left middle and inferior frontal gyri, ipsilateral to the anode electrode (with the cathode over the right supraorbital area), similar to our results, but increased in regions contralateral to the anode electrode (or ipsilateral to the cathode electrode). Others reported increased rs-fcMRI in bilateral OFC (Leaver et al., 2022) and in frontal regions ipsilateral to the anode electrode (Keeser et al., 2011). Little is known regarding frontal networks containing strictly the DLPFC and OFC in rs-fcMRI in healthy populations. However, their interactions via separate networks are associated with cognitive control. The frontoparietal (central executive) network is anchored in the DLPFC, supports executive functions, and integrates information from other networks such as the default mode network (Vincent et al., 2008; Uddin et al., 2019). Anticorrelated rs-fcMRI between these two networks is associated with better cognitive functioning in healthy individuals (e.g., Hampson et al., 2010; Keller et al., 2015; Ng et al., 2016). Regions within the default mode network might also be worth targeting with tDCS such as the precuneus which may modulate rs-fcMRI of key executive control regions (e.g., DLPFC). It will also be of interest to investigate whether repeated tDCS sessions engage these networks and enhance cognitive functions associated with these networks, such as rs-fcMRI of the fronto-parietal network known to be related with attentional processes (Seeley et al., 2007).

There were also changes with sham stimulation involving two of these networks, the fronto-parietal and the fronto-parietooccipital networks. Specifically, rs-fcMRI between the frontal and parietal network decreased after sham stimulation, which was not predicted by baseline rs-fcMRI. Also, rs-fcMRI between the frontal and parietooccipital network increased during sham, but decreased after sham stimulation. Several studies also reported changes in rs-fcMRI involving the DLPFC with sham tDCS, especially implicating the parietal cortex (Peña-Gómez et al., 2012; Mondino et al., 2020; Leaver et al., 2022), the primary auditory association cortex (Peña-Gómez et al., 2012), and the cerebellum (Park et al., 2013). Interestingly, Leaver et al. (2022) compared sham tDCS with a no-tDCS condition. They observed significant decreased rs-fcMRI for the no-tDCS condition as compared to sham tDCS in several networks with the DLPFC, including the superior parietal lobule, the posterior cingulate cortex, the dorsal anterior cingulate, the primary visual cortex, the primary auditory cortex, and the primary somatosensory cortex. We previously discussed that such decrease as we observed in fronto-parietal networks may be linked to a time effect considering that participants stayed at a resting state for more than half an hour, in which DLPFC activity may weaken and decorrelate from the other nodes of resting state networks (as observed in healthy individuals, Mondino et al., 2020). Although rs-fcMRI research has rapidly grown over the last two decades, several questions remain to be addressed. Resting-state networks are generally stable, however, some studies reported that rs-fcMRI does not remain static and fluctuates with time during scanning sessions (Hutchison et al., 2013; Preti et al., 2017; Lurie et al., 2020). This becomes even more pertinent for repeated rs-fcMRI scans within the same scanning period as in our work, such as collecting rs-fcMRI for 5–10 min before tDCS, for 25–30 min during tDCS, and for 5–10 min after tDCS (typically limiting the entire MRI session to 1 h). Hence, this highlights the importance of including no-tDCS conditions, as well as further characterizing rs-fcMRI during repeated acquisitions.

The electrical current travels from the anode to the cathode electrode. However, it is important to highlight here that tDCS did not modulate rs-fcMRI between the regions under the anode and cathode electrodes, here the left and right DLPFC, during or after stimulation. This lack of rs-fcMRI changes was also observed in previous studies applying the tDCS electrodes over both DLPFCs (Mondino et al., 2020), over the left DLPFC and contralateral supraorbital area (Keeser et al., 2011; Peña-Gómez et al., 2012; Park et al., 2013), and over the left DLPFC and contralateral ventrolateral PFC (Leaver et al., 2022). These findings compel us to be cautious when interpreting the impact of tDCS on cognition or behaviors as solely due to brain activity changes in regions under the electrodes, at least when applied over the DLPFC. Likewise, it is tempting to speculate how findings from this work are relevant for clinical populations since the DLPFC is among, if not, the most targeted region with tDCS, especially psychiatric disorders (Fregni et al., 2021). However, it is often expected that patients display different rs-fcMRI as compared to healthy controls (Kaiser et al., 2015; Taebi et al., 2022), thus the tDCS effects on rs-fcMRI might be different in patients from those in healthy individuals.

This study has limitations that should be addressed, such as the small sample size, which limits generalizability. Despite this, we strictly controlled for type 1 error, which should help power analyses for future studies. Also, potential sex-related differences were not studied, which could be examined with an appropriate power analysis in future work. Additionally, the scan time during stimulation was longer than the scanning durations before and after stimulation, which may reduce reliability (Birn et al., 2013). It may be interesting to compare different rs-fcMRI times (e.g., 5-min increments) in future work. To note, in our previous concurrent tDCS-rs-fcMRI study, there were no significant differences in tDCS-induced effects on rs-fcMRI changes in fronto-parietal circuitry when comparing two 15-min time bins during 30 min of tDCS in healthy individuals (Mondino et al., 2020).

In sum, tDCS delivered over the bilateral DLPFC modulates rs-fcMRI of several circuits comprising regions distal (parietal, occipital, temporal) and proximal (frontal) to the anodal electrode, both during and after stimulation. Further, rs-fcMRI prior to tDCS predicted tDCS effects during and after stimulation, which may be useful to identify best tDCS responders in future work.

## Data availability statement

The raw data supporting the conclusions of this article will be made available by the authors, without undue reservation.

## Ethics statement

The studies involving human participants were reviewed and approved by the CIUSSS de la Capitale-Nationale. The patients/participants provided their written informed consent to participate in this study.

## Author contributions

SF designed the study. SF and ER collected data. AB led data analysis with the participation of ER. AB led the interpretation of results with the participation of SF. AB wrote the first draft of the manuscript. All authors critically reviewed the manuscript and approved the finalized version.

## Footnotes

1
https://www.nitrc.org/projects/artifact_detect

2
https://www.nitrc.org/projects/rex/
